# The HER2-Binding Affibody Molecule (Z_HER2∶342_)_2_ Increases Radiosensitivity in SKBR-3 Cells

**DOI:** 10.1371/journal.pone.0049579

**Published:** 2012-11-14

**Authors:** Lina Ekerljung, Johan Lennartsson, Lars Gedda

**Affiliations:** 1 Department of Radiology, Oncology and Radiation Sciences, Division of Biomedical Radiation Sciences, Rudbeck Laboratory, Uppsala University, Uppsala, Sweden; 2 Ludwig Institute for Cancer Research, Uppsala University, Uppsala, Sweden; 3 Swedish Radiation Safety Authority, Research and International Co-operation, Stockholm, Sweden; The Chinese University of Hong Kong, Hong Kong

## Abstract

We have previously shown that the HER2-specific affibody molecule (Z_HER2∶342_)_2_ inhibits proliferation of SKBR-3 cells. Here, we continue to investigate its biological effects *in vitro* by studying receptor dimerization and clonogenic survival following irradiation. We found that (Z_HER2∶342_)_2_ sensitizes the HER2-overexpressing cell line SKBR-3 to ionizing radiation. The survival after exposure to (Z_HER2∶342_)_2_ and 8 Gy (S_8Gy_ 0.006) was decreased by a factor four compared to the untreated (S_8Gy_ 0.023). The low HER2-expressing cell line MCF-7 was more radiosensitive than SKBR-3 but did not respond to (Z_HER2∶342_)_2_. Treatment by (Z_HER2∶342_)_2_ strongly increased the levels of dimerized and phosphorylated HER2 even after 5 minutes of stimulation. The monomeric Z_HER2∶342_ does not seem to be able to induce receptor phosphorylation and dimerization or sensitize cells to irradiation.

## Introduction

The tyrosine kinase receptor HER2 (ErbB2/neu) is one of four members of the epidermal growth factor receptor (EGFR) family. Abnormal expression and signaling of the receptor is associated with development and progression of several forms of cancer, and is also associated with enhanced invasiveness and resistance to chemotherapy and radiation [1], [2]. This makes it an important cell-surface structure for development of targeting agents, both for therapy and imaging, or as a prognostic biomarker for e.g. trastuzumab therapy [3].

While the other members of the EGFR family (EGFR, HER3 and HER4, also denoted as ErbB1-4) can be bound by many different growth factors (e.g. EGF and neuregulins), HER2 does not have any natural ligand. Nevertheless, HER2 is known as the most potent receptor and the preferred dimerization partner [4], [5]. Activation of the receptors occurs through hetero- or homodimerization with another member of the EGFR family, resulting in trans-phosphorylation of tyrosine residues in the intracellular part of the receptor. These phosphorylation sites serve as initiation points for various signaling pathways leading to cellular processes such as proliferation, migration and apoptosis. The effect on downstream signaling, and hence the biological outcome, depends on the composition of the receptor pair and the identity of the ligand [5].

Affibody molecules (Affibody®) are based on the 58 amino acid bundle of the Z domain of staphylococcal protein A. They are usually generated by phage display-based selection from libraries where 13 surface-exposed amino acids have been randomized. High affinity binders to a variety of proteins, e.g. insulin, EGFR and Amyloid-β, have been identified [6] and an imaging study on breast cancer patients showed promising results [7]. In a previous study, we have shown that (Z_HER2∶342_)_2_ inhibits proliferation of SKBR-3 [8].

In this study we investigated the effect of the HER2-binding affibody molecule, (Z_HER2∶342_)_2_ in combination with external γ-radiation and found that (Z_HER2∶342_)_2_ sensitizes SKBR-3 cells to radiation. We have also studied (Z_HER2∶342_)_2_ ‘s ability to induce receptor phosphorylation and dimerization.

## Materials and Methods

### Cell Lines

The human breast cancer cell lines SKBR-3 and MCF-7 were purchased from ATCC (American Type Culture Collection, Rockville, MD, USA). SKBR-3 cells express approximately 2–6×10^6^ HER2 and 10^5^ EGFR receptors per cell [9], [10]. MCF-7 cells express low levels of both EGFR and HER2, about 10^4^ receptors per cell, and high levels of HER3 [11].

SKBR-3 and MCF-7 cells were cultivated in RPMI1640 culture medium supplemented with 10% fetal bovine serum (FBS), 2 mM L-glutamin, 100 IU/ml penicillin and 100 µg/ml streptomycin (Biochrom KG, Germany). For MCF-7, Non-Essential Amino Acids (1×) and Sodium Pyruvat (1 mM) were also added to the culture medium.

### Reagents

The HER2-specific antibody was from Santa Cruz Biotechnology (Santa Cruz, USA), and the EGFR specific antibody from Cell Signaling Technology (Boston, USA). The antibody directed to β-actin was from Sigma-Aldrich (Saint Louis, USA). Anti-mouse and anti-rabbit antibodies linked with horseradish peroxidase were purchased from Invitrogen (Paisley, UK). The affibody molecules, Z_HER2∶342_ and (Z_HER2∶342_)_2_, were kindly provided by Affibody AB (Bromma, Sweden). Epidermal growth factor (EGF) was from Millipore (Billerica, USA) and neuregulin (NRG1-β1) from R&D systems (Minneapolis, USA). DuoSet® ELISA development kits, which measure phosphorylated EGFR, HER2 and HER3, were also bought from R&D Systems.

### Binding Assay

Twenty MBq of ^125^I was added to 5 µl of SPMB (N-succinimidyl p-(trimethylstannyl) benzoate 1 mg/ml 5% acetic acid in methanol). Radio-labelling was initiated by adding 20 µl of Chloramine-T (2 mg/ml, 5% acetic acid in MeOH) and mixed for 5 min. The labelling reaction was terminated by adding 40 µl NBS (sodium metabisulphite, 2 mg/ml in dH_2_O), followed by addition of 50 µg of (Z_HER2∶342_)_2_ (0.2 µg/µl in 0.1 M borate buffer pH 9). The coupling reaction continued for 1 h with continuous shaking. The reaction mixture was separated on NAP-5 column and the high molecular weight fraction containing ^125^I-(Z_HER2∶342_)_2_ was eluted in 1 ml according to the manufacturer.

The binding of ^125^I-(Z_HER2∶342_)_2_ to cultured SKBR-3 cells was monitored in real-time at room temperature using LigandTracer Grey (Ridgeview Instruments, Uppsala, Sweden) [12]. Increasing concentrations of ^125^I-(Z_HER2∶342_)_2_ in culture medium were added in an affinity assay. Cells were incubated for 5 hours for each concentration. The off-rate was measured overnight. Data evaluation and estimation of the kinetic parameter K_D_ were performed using the software TraceDrawer 1.3 (Ridgeview Instruments) using a one-to-one binding model with depletion correction.

### Receptor Phosphorylation and Dimerization Assays

Cell lysis and western blotting were performed as previously described [13]. All antibodies were used according to the manufacturer’s instructions in PBS-T (Phosphate Buffered Saline-Tween 20) with 1% BSA and 0.1% NaAzide. For the dimerization study, the cells were washed with PBS and then incubated with 5 mM BS^3^ (Bis(Sulfosuccinimidyl) suberate, Thermo Fisher Scientific Inc. Rockford, USA) in phosphate buffer, pH 8, for 30 min at room temperature. To end the crosslinking reaction, Tris-HCl was added to a final concentration of 20 mM and set on the bench for 15 min. After this, the cells were lysed, as previously described, and subjected to ELISA or SDS-PAGE (3–8% Tris-Acetate gels from Invitrogen), followed by western blot.

### Survival Assays

For the clonogenic survival, sub-confluent cultures of SKBR-3 or MCF-7 cells were treated with 10 nM (Z_HER2∶342_)_2_ or left untreated for 2–24 hours at 37°C. Thereafter, the cells were irradiated with ^137^Cs γ-ray photons (Gammacell 40 Exactor, MDS Nordion, Kanata, Canada) at a dose rate of 1.034 Gy/min. The total dose was 2, 6 or 8 Gy. The cells were allowed to repair for 16 hours at 37°C before being trypsinized and reseeded at suitable concentrations. The cells were cultured for a time that allowed colony formation, 2–3 weeks depending on the growth rate of the cell line. The cells were fixated in 97% ethanol and then stained with hematoxylin. Colonies with more than 50 cells were counted. In the growth extrapolation method [14], SKBR-3 cells exposed to external γ-radiation were followed until exponential growth was reached. This time, treatment with monomeric Z_HER2∶342_ was also included. The cells were reseeded directly after irradiation.

### Statistical Methods

For the survival assays, GraphPad Prism 4 (GraphPad Software, Inc., San Diego, USA) was used to perform an unpaired t-test. A *P*-value <0.05 was considered significant.

## Results

### Binding Assay

**Figure 1:**
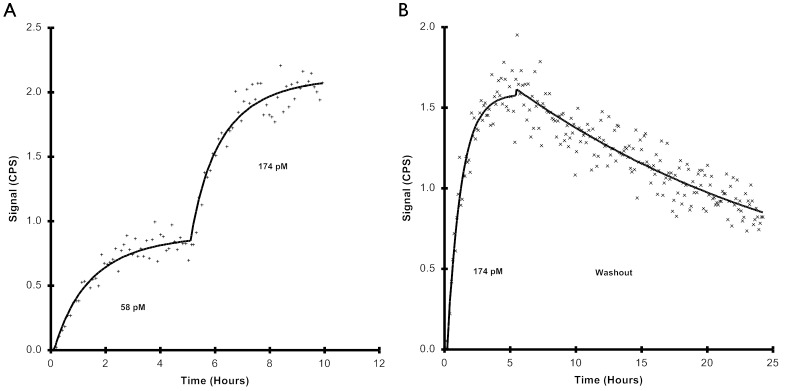
The binding trace of ^125^I-(Z_HER2∶342_)_2_ to SKBR-3 cells. The interaction was monitored in real-time at room temperature using LigandTracer Grey. A) In the first experiment two concentrations of ^125^I-(Z_HER2∶342_)_2_ were added after each other. First, cells were incubated with 58 pM (Z_HER2∶342_)_2_ and thereafter, when equilibrium had been reached, more substance was added to a total concentration of 174 pM. B) In the second experiment, the off-rate after equilibrium of 174 nM exposure was followed for 24 hours. Data evaluation and estimation of the kinetic parameter K_D_ were performed using the software TraceDrawer, using a one-to-one binding model with depletion correction. CPS (Counts per second).

To verify that (Z_HER2∶342_)_2_ binds to SKBR-3 cells, real-time binding of ^125^I-labelled affibody was measured using LigandTracer. Cell binding at two different concentrations and retentions were measured. As can be seen in Figure 1A, (Z_HER2∶342_)_2_ shows an increasing binding trace with increasing concentration. Using these data, in combination with data acquired from off-rate at the higher concentration (Figure 1B), kinetic evaluation of (Z_HER2∶342_)_2_ was estimated with TraceDrawer. The K_D_ was calculated to 6 pM, using a 1∶1-binding model. Due to limitations in the assay, concentrations that rendered ligand depletion were used. However, during kinetic evaluation the software could adjust for ligand depletion and the obtained curve fit conformed well with measured data.

### Receptor Dimerization and Phosphorylation

**Figure 2:**
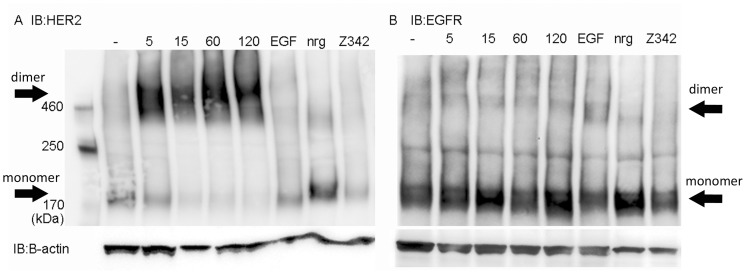
Receptor dimerization. SKBR-3 cells were treated with 10 nM (Z_HER2∶342_)_2_ for 5, 15, 60 and 120 minutes. Z_HER2∶342_ was used at 100 nM for 1 h. Untreated cells (−), and cells treated with EGF and nrg1-β1 for 15 minutes were used as controls. The SKBR-3 cells were cross-linked by 5 nM BS^3^ for 30 minutes before cell lysis. Total cell lysates were then subjected to SDS-PAGE and western blot with antibodies specific for HER2 (A) and EGFR (B). (Z_HER2∶342_)_2_ treatment resulted in dimerization of HER2 even after 5 minutes of incubation. Numbers indicate minutes of stimulation.

**Figure 3:**
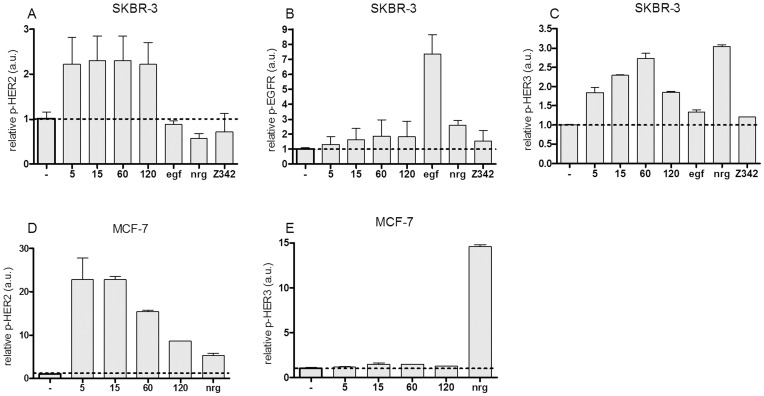
Phosphorylated levels of EGFR, HER2 and HER3, as measured by ELISA. The relative levels for the treated samples were determined by comparing those with the untreated control (−) which was arbitrarily set as 1. Numbers indicate minutes of stimulation by (Z_HER2∶342_)_2_. EGF and NRG1-β1 was used for 5 minutes. A–C) SKBR-3 cell line. D–E) MCF-7 cell line. Mean values and standard deviation from at least two independent measurements are presented.

To determine if binding of (Z_HER2∶342_)_2_ could influence the receptor activation state, we investigated the phosphorylation and dimerization of HER2. Since HER2 can form heterodimers with other members of the EGFR family, we also studied EGFR and HER3. Expression of HER4 is too low in the current cell lines to be of interest [11]. Analysis of dimerization was done in SKBR-3 and MCF-7 cells by crosslinking proteins with BS^3^ (Bis[Sulfosuccinimidyl] suberate) before cell lysis. The monomeric and dimeric receptors were then separated by SDS-PAGE and detected by western blot. As can be seen in Figure 2A, lane 2–5, treatment by (Z_HER2∶342_)_2_ induce dimerization of HER2 in SKBR-3 cells. In contrast, treatment with the monomeric affibody molecule, Z_342_, did not result in any receptor dimerization (lane 8). Except for a tendency for increased dimeric EGFR upon EGF stimulation, no large effects on EGFR dimerization could be detected (Figure 2B). HER3 was only detected in monomeric form in SKBR-3 cells (data not shown). In MCF-7 neither EGFR nor HER2 could be detected and HER3 was only detected in monomeric form (data not shown). Phosphorylation of the receptors was measured by ELISA. Unstimulated cells had low baseline of phosphorylated EGFR, HER2 and HER3. The only exception was as expected high levels of p-HER2 in SKBR-3. In accordance with a previous result by western blot [8], HER2 was phosphorylated by (Z_HER2∶342_)_2_ in SKBR-3 cells (Figure 3A). This was also seen in MCF-7 cells, although decreased over time (Figure 3D). HER2 was not phosphorylated by EGF or NRG1-β1 in SKBR-3. In MCF-7, NRG1-β1 activated HER2 but not to the same extent as (Z_HER2∶342_)_2_. As shown in Figure 3B, no significant difference in the levels of phosphorylated EGFR could be seen for SKBR-3 when incubated with (Z_HER2∶342_)_2_. EGFR was stimulated only by EGF (10 nM for 5 min). For MCF-7 cells, the levels of p-EGFR were too low to draw any conclusions (data not shown). Treatment with (Z_HER2∶342_)_2_ phosphorylated HER3 in SKBR-3 but not in MCF-7 cells (Figure 3C and E). Stimulation with 10 nM of NRG1-β1 for 5 minutes was used as a positive control for phosphorylation of HER3 (Figure 3C and E).

### Survival Assays

**Figure 4:**
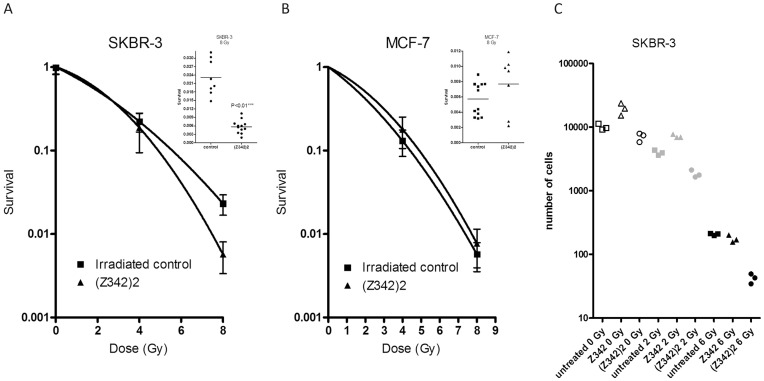
Survival after exposure to ionizing radiation. A–B) Clonogenic survival. Cells were treated with 10 nM (Z_HER2∶342_)_2_ for 2 hours prior to irradiation and allowed to recover for 16 hours before reseeding. Mean values and standard deviations are calculated on at least 7 replicates. The linear quadratic model was used for curve fitting and an unpaired t-test to test for significance. A) SKBR-3. Inset; after irradiation with 8 Gy the cells treated with (Z_HER2∶342_)_2_ had a significantly lower (*P*<0.01) survival than the control group. B) MCF-7 cells. Inset; after irradiation with 8 Gy the cells treated with (Z_HER2∶342_)_2_ did not differ from the control group. C) Growth extrapolation method. SKBR-3 cells were treated with 16.6 nM (Z_HER2∶342_)_2_, Z_HER2∶342_ or left untreated for two hours, irradiated and then reseeded. The figure shows the number of cells normalized to the starting value in each group after 28 days of cultivation in normal cell culture medium.

To investigate if (Z_HER2∶342_)_2_ could sensitize breast cancer cells to ionizing radiation we analyzed clonogenic survival on SKBR-3 and MCF-7 cells. As shown in Figure 4A, treatment of SKBR-3 cells with 10 nM (Z_HER2∶342_)_2_ decreased survival 4-fold after irradiation with 8 Gy compared to the irradiated control (S_8Gy_ 0.006 and 0.023 respectively, *P<*0.01). After 4 Gy of radiation, the two groups did not significantly differ. For MCF-7 cells, treatment with (Z_HER2∶342_)_2_ did not alter the survival, neither at 4 nor 8 Gy (Figure 4B). Survival for the non-irradiated control, plating efficiency (PE), was low for the SKBR-3 cell line; PE (Z_HER2∶342_)_2_ 0.05 and PE (control) 0.09, compared to PE (Z_HER2∶342_)_2_ 0.37 and PE (control) 0.38 for MCF-7 cells.

To verify the results, a different survival model was also used. Growth of SKBR-3 cells was followed after irradiation. This time, treatment with monomeric Z_HER2∶342_ was also included. Figure 4C shows the number of cells after 28 days of cultivation after irradiation by 0, 2 and 6 Gy. In addition a clear effect of (Z_HER2∶342_)_2_ was seen with this assay. At both doses the survival of (Z_HER2∶342_)_2_-treated cells was significantly lower. Extrapolation of the curve fits based on the data points where exponential growth has been reached, results in an estimate of the surviving fraction of cells that are responsible for the regrowth [14]. The surviving fraction calculated by this method was similar to the result from the clonogenic survival, rendering a significant decrease in survival compared to the control (S_6Gy_ 0.002 for (Z_HER2∶342_)_2,_ compared to S_6Gy_ 0.02 for the control (*P*<0.01)). Notably, the monomeric Z_HER2∶342_ did not sensitize for radiation, on the contrary there seemed to be a protective effect at the lower dose.

It should be noted that MCF-7 cells are more radiosensitive than SKBR-3 cells, mean survival after 8 Gy was 0.6% compared to 2.3%. It has been suggested that increased HER2 expression is correlated with radioresistance [15], [16].

## Discussion

The HER2-binding affibody molecule (Z_HER2∶342_)_2_ clearly binds to SKBR-3 cells (Figure 1). The K_D_ was calculated to be 6 pM using a 1∶1 fitting model. Although it is unclear whether this is the best model for a dimeric binder as (Z_HER2∶342_)_2,_ it rendered a good fit and gives a reasonably accurate value of K_D_. Further, the obtained affinity value was, as expected, slightly stronger than the affinity of Z_HER2∶342_, which has a K_D_ of 22 pM (previously shown by Orlova *et. al*. [17]).

The basal level of phosphorylated HER2 was high and dimeric HER2 could be detected even in unstimulated SKBR-3 cells. Since HER2 is known to be able to spontaneously form homodimers when overexpressed [18], [19] and SKBR-3 cells have as many as 2–6×10^6^ receptors per cell [9], [10], the high background level is not surprising. Treatment by (Z_HER2∶342_)_2_ induced dimerization of HER2 in SKBR-3 cells as early as 5 minutes post-treatment and the signal remained high even after 2 hours (Figure 2A). The smearing appearance of the HER2 bands in the dimeric state is commonly seen with tyrosine kinase receptors and may relate to heterogenous glycosylation of the HER2 proteins and possibly other types of post-translational modifications. In addition, we cannot exclude the exitstence of receptor trimers or other higher order complexes. As shown previously [8], (Z_HER2∶342_)_2_ promotes phosphorylation of HER2 (Figure 3A). Surprisingly, HER3 was also phosphorylated in SKBR-3 cells by (Z_HER2∶342_)_2_. Even though no dimeric HER3 could be detected, the phosphorylation must have been induced by heterodimerization, since HER3 has deficient kinase activity [20]. Probably the level of dimeric HER3 was too low to be detectable by western blot, while ELISA, which is a more sensitive method, could detect phosphorylated receptor. The same difference in terms of sensitivity of the methods was seen for HER2 in the MCF-7 cell line, where increased levels of phosphorylated HER2 could be detected by ELISA (Figure 3D), but no HER2 receptor could be detected by western blot at all (data not shown).

Even though HER2 is known as the preferred dimerization partner for ErbB receptors [4], treatment with neither of the natural ligands, the EGFR-binding EGF or the HER3-binding NRG1-β1, markedly increased dimerization or phosphorylation of HER2 in SKBR-3 cells. This might be explained by the high basal activation of HER2 together with the differences in receptor number. Since there are much fewer EGFR and HER3 than HER2, the increase in dimerization and phosphorylation due to heterodimerization might not be detectable amongst the relatively high background. In the low HER2-expressing cell line MCF-7, the basal level of phosphorylated HER2 was very low and could be much increased by stimulation with NRG1-β1.

Treatment with (Z_HER2∶342_)_2_ for as short a time as 2 hours before irradiation decreased survival in SKBR-3 cells by a factor of four at 8 Gy when compared to gamma irradiation only. The radiosensitizing effect of (Z_HER2∶342_)_2_ was also demonstrated in the additional survival analysis where cell growth was followed for several weeks after irradiation. This resulted in a similar decrease in survival compared to the control, S_6Gy_ 0.002 compared to S_6Gy_ 0.02 (*P*<0.01). It should be noted that the growth extrapolation method generally results in lower survival, as shown before [21]. Other bivalent binders that target the EGFR-family have also been shown to sensitize to irradiation, for example trastuzumab and cetuximab [15], [22]. The mechanism for this action is not yet fully understood. However, it has been shown that the repair process for radiation-induced DNA damage can be effected by the EGFR-receptors, both by the receptor itself and also through its downstream signaling effectors phophatidylinositol 3 kinase (PI3K)/Akt and mitogen-activated protein (MAP) kinases/Erk [23], [24]. We have previously shown that the affibody molecules, trastuzumab and cetuximab can effect the level of phosphorylated Akt and Erk [8], [13], [25]. Thus, it is possible that the radiosensitizing effects of the HER2-binding agents are conferred through these signaling pathways. Since the effects of the monomeric Z_HER2∶342_, and the dimeric (Z_HER2∶342_)_2_ differ so much in terms of radiosensitizing, receptor dimerization and phosphorylation, and as we have previously shown, with regards to proliferation and downstream signaling [8], [13], it is possible that the dimeric (Z_HER2∶342_)_2_ can simultaneously bind two HER2 receptors and thereby induce homodimerization. Ligand-induced dimerization is common for the tyrosine kinase receptors, but for the EGFR family dimerization is considered as entirely receptor mediated. As a result of binding of a ligand, the receptor undergoes conformational changes that open up a dimerization arm and thus enable the receptor to dimerize [26]. HER2, on the other hand, has a dimerization arm that is always open [27], so (Z_HER2∶342_)_2_ could in principle span the dimer interface and induce homo-dimerization by pulling two receptors together. (Z_HER2∶342_)_2_ binds to the junction of domain III and IV on HER2, but not to the same site on domain IV as Trastuzumab [28].

To conclude, we have shown that the HER2-binding affibody molecule (Z_HER2∶342_)_2_ significantly decreases survival after γ-irradiation. This radiosensitizing effect makes (Z_HER2∶342_)_2_ interesting for therapy purposes.
